# Parasite species co-occurrence patterns on *Peromyscus*: Joint species distribution modelling

**DOI:** 10.1016/j.ijppaw.2020.04.011

**Published:** 2020-06-14

**Authors:** Jasmine S.M. Veitch, Jeff Bowman, Albrecht I. Schulte-Hostedde

**Affiliations:** a Department of Biology, Laurentian University, 935 Ramsey Lake Road, Sudbury, ON, P3E 2C6, Canada; b Ontario Ministry of Natural Resources and Forestry, Wildlife Research and Monitoring Section, Trent University, 2140 East Bank Drive, DNA Building, Peterborough, ON, K9L 0G2, Canada

**Keywords:** Aggregation, Segregation, Competition, Co-occurrence, Parasite communities

## Abstract

Hosts are often infested by multiple parasite species, but it is often unclear whether patterns of parasite co-occurrence are driven by parasite habitat requirements or parasite species interactions. Using data on infestation patterns of ectoparasitic arthropods (fleas, trombiculid mites, cuterebrid botflies) from deer mice (*Peromyscus maniculatus*), we analyzed species associations using joint species distribution modelling. We also experimentally removed a flea (*Orchopeas leucopus*) from a subset of deer mice to examine the effect on other common ectoparasite species. We found that the mite (*Neotrombicula microti*) and botfly (*Cuterebra* sp.) had a negative relationship that is likely a true biotic species interaction. The flea had a negative association with the mite and a positive association with the botfly species, both of which appeared to be influenced by host traits or parasite life-history traits. Furthermore, experimental removal of the flea did not have a significant effect on ectoparasite prevalence of another species. Overall, these findings suggest that complex parasite species associations can be present among multiple parasite taxa, and that aggregation is not always the rule for ectoparasite communities of small mammals.

## Highlights

• A negative species interaction was identified between a mite and botfly species.
• Additional parasite associations appear to be influenced by host traits or parasite life-history traits.
• Removal of the flea species did not have an effect on prevalence of another species.

## Graphical abstract

**Figure:**
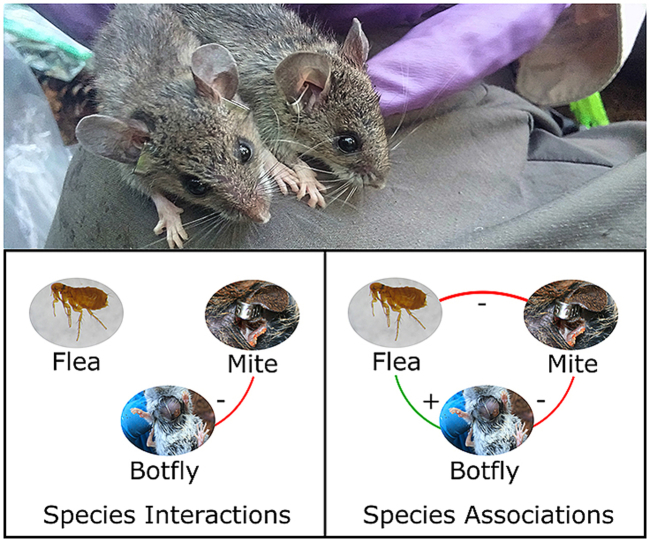

## 1 Introduction

Ecological communities include a complex network of biotic interactions such as predation, parasitism, competition, mutualism and facilitation (Fontaine et al., 2011). Parasites play a fundamental role in these communities, in part because parasites represent a substantial portion of worldwide biodiversity and biomass, but also due to the influence parasites can have in shaping biological communities and maintaining biodiversity (Poulin, 1999). It is also important to note community dynamics at a smaller scale. For parasites, hosts can be considered ecosystems that are discretely bound and that have easily defined resource availabilities (host size and abundance) (Rynkiewicz et al., 2015). Hosts are often infested by numerous ectoparasite species and thus, insight might be gained by assessing relationships of multiple parasite species simultaneously (Dargent et al., 2017). Different parasite species can interact either directly (through competition for resources) or indirectly (through modulation of a host's immune system) while on the host (Rynkiewicz et al., 2015).

When investigating parasite assemblages among individual hosts, two patterns might result: (1) species co-occur as often as expected by chance, or (2) species co-occur more or less often than expected by chance (Krasnov et al., 2006a). Random patterns of species co-occurrence adhere to the unified neutral theory of biodiversity, predicting that distributions of species abundances are mainly controlled by ecological drift (i.e. random variation of species abundances where each species has an equal chance of reproduction or death) (Norris, 2003). Population dynamics are mainly influenced by birth, death, immigration, and emigration; however, this may not be the case if there are large differences in functional equivalence of parasite species (ex. endo- and ectoparasites, site selection, food preferences) (Poulin, 2004; Hubbell, 2006; Bellay et al., 2015). If the demographics of parasite species are distinct on a per capita basis (i.e. species are not equivalent to one another), then we can expect divergence from unified neutral theory in a parasite community (Hubbell, 2006).

Non-random patterns in parasite species assemblages are generally due to interspecific competition or environmental conditions, including the host environment (i.e. host characteristics) and external environment (i.e. host population abundance and habitat of the host) (Mouillot et al., 2005; Van den Wyngaert et al., 2014; Dallas et al., 2019). Species that are more similar tend to have a greater magnitude of competition, leading to negative species interactions or competitive exclusion (e.g. Kuris and Lafferty, 1994). External environmental factors can act as filters through which only species with similar habitat requirements will co-exist with one another, leading to positive species interactions (Krasnov et al., 2006a). A similar pattern can be seen with a host environment, such as when hosts with reduced anti-parasitic responses are more susceptible to multiple different parasite species, facilitating co-existence and positive interactions (Krasnov et al., 2005). Furthermore, infection of one parasite species can facilitate infection of another through host deterioration, immunosuppression, or immunological trade-offs (Moreno et al., 2013; Dallas et al., 2019). These mechanisms are not mutually exclusive (Mouillot et al., 2005), though it is not yet clear which mechanism has the largest effect on community structure in ectoparasites of small mammals.

While many studies have explored community dynamics of parasites through observational data, investigations into the consequences of removing a species on parasite community dynamics is rarely undertaken (Fenton et al., 2014). Experimental addition or removal of species can provide insight into community stability and the effects of a species on community structure (Knowles et al., 2013; Pedersen and Antonovics, 2013). For parasite communities, this can be explored through perturbation experiments using an insecticide treatment on a host to reduce or remove a target parasite species (Rynkiewicz et al., 2015). Perturbation experiments are the most reliable method to assess species interactions and are essential to determine critical parasite species interactions that may affect drug treatments and host health (Fenton et al., 2014).

Parasite species interactions are well documented in laboratory studies, however similar studies on wild populations are often observational, leading to confounding factors with processes unrelated to species interactions (Knowles et al., 2013; Hoffmann et al., 2016). In contrast, studies combining longitudinal monitoring of parasites and experimental removal of targeted parasite species are rare but more appropriate for identifying interspecific relationships that are not always evident through observational studies (Lutermann et al., 2015; Hoffmann et al., 2016). Perturbation experiments have provided important insights on parasite community stability, long-term carry-over effects of perturbations and the influence of parasite life-history on perturbation responses (Knowles et al., 2013; Lutermann et al., 2015; Hoffmann et al., 2016).

Overall, interspecific interactions in parasite communities have remained largely understudied, with the majority of literature focusing on endoparasites, and studies that do examine ectoparasites mainly focusing on interactions within the same taxa (Hoffmann et al., 2016). Recent perturbation studies on both endo- and ectoparasites have identified the presence of mainly competitive interactions between parasite taxa (Knowles et al., 2013; Lutermann et al., 2015; Hoffmann et al., 2016). However, facilitative interactions are more common in studies of ectoparasites on rodents (Krasnov et al., 2010; Nava and Lareschi, 2014). Strong interactions appear to mainly occur between species sharing feeding sites, though parasite species are also known to compete for food resources or through modulation of the host's immune system (Pedersen and Fenton, 2007; Knowles et al., 2013; Hoffmann et al., 2016). Therefore, targeted removal of an ectoparasite species and examination of other parasite species from different taxa may provide a clearer understanding of interspecific interactions among ectoparasite species.

We examined patterns of co-occurrence in ectoparasitic arthropods (fleas, trombiculid mites, cuterebrid botflies) of deer mice (*Peromyscus maniculatus*) to determine whether ectoparasite species co-occur more or less often than expected by chance, and whether any observed non-random associations were positive or negative. We expected that non-random patterns would follow an aggregative structure with mainly positive associations, consistent with previous studies on ectoparasites of rodent hosts (Krasnov et al., 2010, Nava and Lareschi, 2014, Colombo et al., 2015, Colombo et al., 2015, but see Hoffmann et al., 2016). We also expected that if non-random co-occurrences were observed with target ectoparasite taxa (fleas), experimental removal would significantly alter prevalence of other ectoparasite species.

## 2 Materials and methods

### 2.1 Study area and trapping

This study was conducted in Algonquin Provincial Park, Ontario, Canada (45°54′ N, 78°26’ W) from May to August of 2017 and 2018. Ectoparasites of deer mice (*Peromyscus maniculatus*), were sampled across seventeen 100-m traplines with two Sherman live traps (H. B. Sherman Traps, Inc., Tallahassee, Florida) placed at each station, 10-m apart (detailed methods in Fryxell et al., 1998). Trapping occurred over 3 consecutive days in two-week periods. Sampling of deer mouse ectoparasites was conducted throughout a variety of habitats, including sugar maple hardwood, cut-over mixed-wood, dense mixed-wood, conifer, white pine and white spruce, black spruce and aspen, white pine and red pine and a pine plantation.

Captured deer mice were transferred to a handling bag, sexed and assessed for reproductive status (non-reproductive, scrotal, perforated, pregnant, lactating). Individuals were weighed using a Pesola® scale (±0.1 g). Age of deer mice (juvenile, subadult, adult) was categorically determined from body mass and fur colour (Schmidt et al., 2019). Individuals received two metal ear tags with unique alphanumeric codes (National Band and Tag Co., Newport, Kentucky, USA). All methods used were reviewed and approved by the Animal Care Committee (ACC) at Laurentian University, Sudbury, Ontario, Canada, protocol number 2017-06-01.

### 2.2 Visual assessment of parasite specimens

Captured animals were visually examined for fleas (Siphonaptera), trombiculid mites (Trombiculidae), and cuterebrid (Diptera) botflies. We applied systematic searching for ectoparasites, spending 60 s each on the dorsal and ventral side while gently combing the fur backwards with a fine-toothed comb and blowing on the fur (Patterson et al., 2013). Only ectoparasites large enough for visual observation without the need of a microscope were included in this study. While combing and visual assessment in combination is a reliable method for examining fleas, lice and ticks, it is possible that mite species can be missed through visual inspection and combing (Beaumont et al., 2019); therefore, it is possible that we were not able to identify additional mite species in this study. However, this is a common method of quantifying ectoparasite communities on small mammals that has been used in multiple studies (ex. Buchholz and Dick, 2017; Pero and Hare, 2018; Beaumont et al., 2019).

### 2.3 Experimental parasite removal

In 2018, fleas were experimentally removed from some deer mice. Fleas were chosen as the target taxa for removal as they are one of the most common ectoparasites of deer mice and could be removed using an insecticide treatment that would not directly impact prevalence of other ectoparasite species (i.e. mites, botflies). Traplines were paired by habitat type (sugar maple hardwood, cut-over mixed-wood, dense mixed-wood, conifer, white pine and white spruce, black spruce and aspen, white pine and red pine) and were assigned to a control or treatment group. 60 traps were used for the sugar maple hardwood and cut-over mixed-wood habitat types, 40 were used for the dense mixed-wood, conifer, white pine and white spruce, black spruce and aspen, white pine and red pine. As there was only one trapline (with 20 traps) for the pine plantation habitat, this data was not included in the parasite removal analysis.

In the treatment group, fleas were removed from mice through combing and application of 0.8-μL of Frontline Plus (Merial Limited, Duluth, GA, USA, main active ingredient: fipronil 0.29%). All flea specimens were stored in 70% ethanol for future identification. In the control group, mice were visually examined for ectoparasites and did not have their fleas removed and did not receive Frontline Plus. The ectoparasite quantification methods used to compare between the control and treatment group is similar to those previously used on Franklin's ground squirrels (Pero and Hare, 2018). Since Frontline Plus is only effective at preventing infestation of fleas and ticks (Wiedemann, 2000a,b, Hoffmann et al., 2016), we did not remove the other ectoparasite species. Juvenile deer mice were not treated with Frontline Plus and were excluded from the ectoparasite removal experiment.

### 2.4 Taxonomic identification

A subset of ectoparasites (specifically fleas and mites) were combed from hosts’ fur and collected using tweezers to identify to species. Mite specimens were collected in 2016 prior to data collection for the present study. For flea specimens, we removed all fleas observed in 2017, and all fleas from traplines in the parasite removal treatment group in 2018. As parasitic botfly larvae remain in subcutaneous cavities while infecting hosts, we were not able to remove any botflies in this study. Specimens were stored in 70% ethanol. Recaptures of individuals with ectoparasites removed were not included in the dataset unless at least a week had passed since ectoparasite removal because fleas and mites take ~3–4 days to recolonize a host after parasite removal (unpublished data). Given that ectoparasites were removed over the course of the study, which may influence intensity measures, we only focused on prevalence in our statistical analysis.

The subset of fleas removed from deer mice were prepared for detailed morphological examination and identification using balsam mounts (Richards, 1964). The remaining flea samples were examined using a dissecting microscope (Olympus SZ61). All flea samples were identified to species using Siphonaptera keys (Holland, 1985; Lewis, 2000) and with the assistance of T. Galloway (University of Manitoba, Canada). The mite species was identified to species by H. Proctor (University of Alberta, Canada). As we were unable to collect a botfly larva from subcutaneous tissue to identify to the species level, but given geographical range and host preference, it was likely *Cuterebra fontinella* or *americana* (Stewart, 2011).

### 2.5 Statistical analysis

Many questions in community ecology, including presence and direction of species co-occurrence patterns, require a joint analysis of species presence or abundance data gathered concurrently across different taxonomic groups (such as different species) (Warton et al., 2015; Aivelo and Norberg, 2018). These kinds of datasets often include a large number of zeros, such as studies examining parasite counts (Warton et al., 2015). Furthermore, individual samples may not contain a great deal of information. Joint species distribution models integrate the joint distribution of species, as well as zero-inflated data, allowing investigation of species co-occurrence patterns (Clark et al., 2014). By examining distributions of multiple species simultaneously, these models can be used to study relationships between species that occur due to and regardless of environmental responses (Aivelo and Norberg, 2018).

The ectoparasite screening method determined infestation status (yes or no for each parasite species) at the individual level, and so we considered these results as multiple observations on the same individual. Statistical models focused on species presence/absence observations as units of analysis accordingly, while incorporating higher-level hierarchical effects to account for host individuals. Data from mice on traplines from which fleas were removed were not included in this analysis. Furthermore, only ectoparasites infesting more than 10 host individuals were included to avoid complications due to small sample sizes. Consequently, we used multivariate joint species distribution models to control for host biology and external environment factors while assessing species co-occurrence patterns. We fitted all models with a Bayesian inference by applying the hierarchical modelling of species communities (HMSC) approach using the ‘Hmsc’ package version 2.99–0 (Ovaskainen et al., 2017). HMSC uses a multivariate generalized linear mixed modelling approach that can analyze both small and large datasets while exhibiting a high degree of accuracy (Warton et al., 2015; Ovaskainen et al., 2017; Ovaskainen and Abrego, 2020). Separate models were run for each common ectoparasite species (i.e. >10 host individuals infested). All analyses were conducted using statistical software package R version 3.5.2 (R Core Team, 2018).

For each analysis, we modelled the response parasite co-occurrence matrix (Y matrix), which describes the presence/absence of the ectoparasite species, using a probit model with the default priors of the framework (Ovaskainen et al., 2017). We included data on environmental covariates that are expected to contribute to ectoparasite community composition (X dataframe). Specifically, we included host biology factors: host age (juvenile, subadult or adult), sex, reproductive status (non-reproductive, reproductively active), body mass, and external environment factors: an index of host population abundance (number of host individuals measured as captures per 100 trap nights), and date as fixed effects for the X dataframe. Individual ID number and year were specified as random effects. Analyses for deer mice also included an index of population abundance for additional small mammal species (southern red-backed voles (*Myodes gapperi*), woodland jumping mice (*Napaeozapus insignus*)), which are known to share similar space or food preferences (Vickery, 1979; Boonstra and Krebs, 2012). Thus, conspecific hosts may influence ectoparasite prevalence on deer mice through parasite transmission, but also through resource competition, which can influence immune function (Merlot et al., 2004; Tardy et al., 2018). This index of population abundance was measured as captures per hundred trap-nights at each trapline over two-week periods, as used by Stewart et al. (2014).

We fitted two different models: (1) a model constrained by fixed and random effects, and (2) an unconstrained model with only random effects included, which is akin to comparing a constrained and unconstrained ordination (Aivelo and Norberg, 2018). By comparing results from constrained and unconstrained models, we can investigate whether non-random ectoparasite species co-occurrence patterns are shaped exclusively by (host and external) environmental factors, whether the co-occurrence patterns are hidden by environmental conditions, or whether the interactions are unaffected by our predictor variables (Aivelo and Norberg, 2018). Examining both models allows for investigation of interspecific parasite interactions while accounting for host and external environmental factors, and thus disentangling effects of species interactions, host biology and external environment factors on occurrence patterns. Uninfested hosts were included in the analysis, as uninfested small mammal hosts could be used by arthropod ectoparasites (Krasnov et al., 2006b). Furthermore, through multiple live-captures of the same host individuals, we were able to observe that some uninfested hosts did become infested at later captures, or vice versa (unpublished data). Therefore, inclusion of uninfested hosts was important for this analysis.

Models were fitted by running two Markov chain Monte Carlo (MCMC) chains with 15,000 iterations (first 5000 being burn-in) and thinning remaining iterations by 10. The model was cross-validated using a two-fold cross validation. All effective sample sizes of the parameters were >2000. MCMC convergence was assessed through distributions of potential scale reduction factors (PSRF) for model parameters. Model performance was examined using Tjur's R^2^ and area under the receiver operating characteristic (AUC). Tjur's coefficient of determination (R^2^) provides a measure on the amount of variation the model explains for each species and quantifies the overall ability for the constrained and unconstrained models to explain the occurrences of individual ectoparasite species (Tjur, 2009; Löfgren et al., 2020). The receiver operating characteristic curve plots the relationship between sensitivity (the true positive rate) against the false positive rate. The area underneath is used to assess the discriminatory capacity of species distribution models (Jiménez-Valverde, 2012).

Variance partitioning can be used to assess explanatory power of various predictor variables, therefore highlighting their relative importance in structuring an ectoparasite community (Ovaskainen et al., 2017). The variance in the constrained model was partitioned based on fixed and random effects using the ‘Hmsc’ package version 2.99–0 (Ovaskainen et al., 2017). To provide insights into relative differences in the influence of fixed and random effects, we separated the variance explained among all of the fixed and random effects.

We also used generalized linear mixed-effects models to further examine positive or negative relationships of ectoparasite prevalence with important host biology (host age (juvenile, subadult or adult), sex, reproductive status (non-reproductive, reproductively active), body mass) and external environment (host population abundance, southern red-backed vole population abundance, woodland jumping mouse population abundance, and date) factors from variance partitioning to provide insights into differences between constrained and unconstrained models. Habitat type was not included in this analysis, as it was too complex for the models to perform properly. The focal response variable was individual infestation status (prevalence). Separate models were run for each common ectoparasite species. Statistical analysis of these models was completed using the ‘glmmTMB’ package version 0.2.3.0 with a Bernoulli response variable and logit link function (Magnusson et al., 2017). Two-level categorical variables (host sex, reproductive status) were coded as binary continuous variables to improve model simplicity. Analyses including host age, a multi-level categorical variable, had a zero-sum contrast incorporated. Continuous predictors were centered and scaled by their mean and standard deviation.

A separate generalized linear mixed-effects model was used to examine effects of flea removal on the mite species only, as there were not enough host individuals infested with the botfly species in this study to examine effect of flea removal. Similar to the aforementioned mixed models, the focal response variable was individual infestation status (prevalence), and statistical analysis was completed using the ‘glmmTMB’ package version 0.2.3.0 with a Bernoulli response variable and logit link function (Magnusson et al., 2017). Treatment group (control or treatment), occasion (pre- or post-treatment) and the interaction between these factors were included as fixed effects and individual ID number as a random effect. A P-value was calculated using a likelihood ratio test with the ‘anova’ function in the ‘stats’ package version 3.6.1.

## 3 Results

We identified one prominent flea species (*Orchopeas leucopus*), a trombiculid mite species (*Neotrombicula microti*), and a botfly (*Cuterebra* sp.) on the deer mouse. The final dataset included 229 deer mice for the joint species distribution models with 713 captures, ranging from 1 to 12 captures for each individual (Table 1). There was a total of 59 deer mice for the perturbation experiment removing the flea, with 144 captures ranging from 1 to 5 captures for each individual. Prevalence of the different ectoparasite species varied from 5.7% to 39.3% (Table 2).

**Table 1:** Age and sex distribution of deer mice (n = 229 individuals). Individuals sampled through multiple age classes are counted in both age classes.

	Sex	
Age	Female	Male
Adult	67	67
Subadult	38	66
Juvenile	42	25

**Table 2:** Number and proportion of deer mice (n = 229 individuals) infested by ectoparasite species. Individuals captured multiple times that were infested by an ectoparasite species in at least one capture are included in count of infested individuals.

Parasite species	Site preference	Diet	Infested individuals/229	Prevalence (%)
Mite (*Neotrombicula microti*)	Mainly host ear pinnae, occasionally on genitals, rarely on chin or coxofemoral joint (Ott-Conn et al., 2015)	Host tissue and tissue fluid (Foley et al., 2013)	57	24.9
Flea (*Orchopeas leucopus*)	a	Host blood (Holland, 1985)	46	20.1
Flea (*Megabothris quirini*)	a	Host blood (Holland, 1985)	6	02.6
Flea (*Megabothris acerbus*)	a	Host blood (Holland, 1985)	2	00.9
Flea (*Epitedia wenmanni wenmanni*)	a	Host blood (Holland, 1985)	1	00.4
Botfly (*Cuterebra* sp.)	Mainly inguinal region in subcutaneous cavity (Cogley, 1991)	Host tissue and tissue fluid (Slansky, 2007)	13	05.7

a Fleas tend to be found across the host's body, alternating between time spent on host and in nesting material (Krasnov et al., 2006b).

**Fig. 1:**
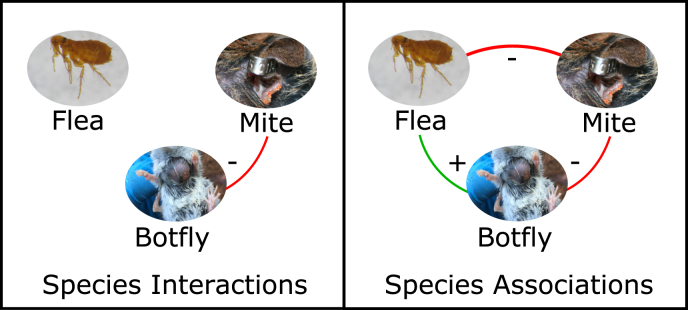
Summary of positive (green, +) and negative (red, -) associations between ectoparasite species (flea (Orchopeas leucopus), mite (Neotrombicula microti), botfly (Cuterebra sp.)) on deer mice with statistical support of at least 95% posterior probability (n = 229 individuals). Results presented are from the constrained model (species interactions) or unconstrained model (species associations). (For interpretation of the references to colour in this figure legend, the reader is referred to the Web version of this article.)

Non-random co-occurrence patterns were identified between ectoparasites of deer mice in both the constrained and unconstrained joint species distribution models (Fig. 1). The unconstrained model contained all identified relationships between species, while the constrained model only contained a single relationship. Specifically, from the constrained model, there was only a negative species relationship between the mite and botfly on deer mice. When examining associations in the unconstrained models, the mite and botfly still had a negative relationship; however, there was also a negative association between the flea and mite, as well as a positive association between the flea and botfly.

**Fig. 2:**
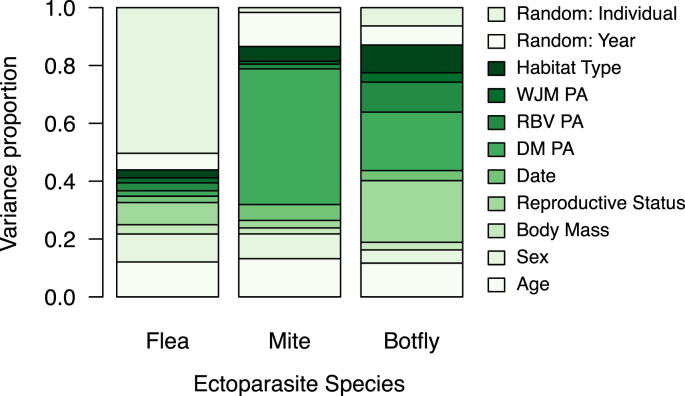
Results of variance partitioning for variation in ectoparasite prevalence explained by fixed and random effects for each ectoparasite species (columns). Explained variance presented for the constrained model for deer mice (n = 229 individuals). DM, deer mice; RBV, southern red-backed vole; WJM, woodland jumping mouse; PA, population abundance. Population abundance of small mammal species measured as captures per 100 trap nights. (For interpretation of the references to colour in this figure legend, the reader is referred to the Web version of this article.)

Variance partitioning of the constrained model for deer mice demonstrated that different ectoparasite species had unique responses to fixed (host age, sex, body mass, reproductive status, date, target and additional host population densities, habitat type) and random (individual, year) effects included in the study (Fig. 2). While the relative importance of variables changed with ectoparasite species, variation explained by fixed effects compared to random effects was lower than 50% for the flea, while it was over 80% for the other species. This was also reflected in the model performance of the ectoparasite species (Supplementary Material Table A1). Host individual ID number explained the largest proportion of variation for fixed and random effects in flea prevalence only, whereas deer mouse population abundance explained the largest proportion of variation for mite prevalence. The distribution of variance explained by fixed and random effects for botfly prevalence was not largely skewed towards a single variable.

Host age, reproductive status, and sex were the most influential fixed effects for the flea in the constrained model (Fig. 2). Host age and sex were also fixed effects of relatively high importance (explained more than 10% of variance) for mite prevalence, whereas host age and reproductive status were associated with botfly prevalence. While the HMSC-based effect estimates did not show significant variation in these factors for the flea species (Figure A1), we were able to further examine these environmental covariates using generalized linear mixed models (package ‘glmmTMB’ version 0.2.3.0, Magnusson et al., 2017). The flea and mite prevalence differed with host age classes, where flea prevalence was highest on adults and juveniles, but mite prevalence was lowest on adults (Table 3). Furthermore, fleas infested more males than females, while the opposite pattern was observed for the mite. There were no similarities for infestation of host age classes between the flea and botfly; however, both species infested reproductively inactive individuals more often than hosts that were reproductively active.

**Table 3:** Relationship between prevalence of flea (Orchopeas leucopus), mite (Neotrombicula microti) or botfly (Cuterebra sp.) on deer mice and influential fixed effects (host age, sex, reproductive status) shared between ectoparasite species (n = 229 individuals).

Response variable	Covariate	β	SE	p
Flea prevalence	Age_Adult_			0.01
	Juvenile	0.349	0.720	
	Subadult	−1.354	0.621	
	Sex_M_	1.234	1.137	0.26
	Reproductive status	−0.064	1.017	0.95
Mite prevalence	Age_Adult_			0.75
	Juvenile	0.229	0.404	
	Subadult	0.040	0.260	
	Sex_M_	−0.277	0.448	0.54
Botfly prevalence	Age_Adult_			0.53
	Juvenile	−1.172	1.778	
	Subadult	1.049	1.152	
	Reproductive status	−0.992	2.926	0.73

Lastly, mite prevalence was higher in the treatment group where fleas were removed, but there was a weak effect size and no significant difference in mite prevalence between the control and treatment group (Table 4). Furthermore, while mite prevalence increased from pre-to post-treatment in both the control and treatment groups (i.e. mite prevalence increased over time), there was again a weak effect size and no significant difference between the two groups.

**Table 4:** Effect of experimental removal of fleas on mean mite prevalence in a deer mouse population (n = 229 individuals). Treatment indicates mice in control (no flea removal) or treatment group (with fleas removed using Frontline Plus). Occasion indicates pre- and post-treatment. For the control group, pre- and post-treatment represents a ~two-week period. For the treatment group, pre-treatment represents before experimental removal of the flea (Orchopeas leucopus) and post-treatment represents hosts sampled ~ two weeks after experimental removal of fleas.

Covariate	β	SE	p
Treatment	0.166	0.324	0.604
Occasion	0.361	0.295	0.227
Treatment x Occasion	−0.347	0.296	0.225

## 4 Discussion

### 4.1 Model comparison of ectoparasite co-occurrence patterns of deer mice

A single relationship between ectoparasite species was observed in both the constrained and unconstrained models, while two additional relationships were identified in the unconstrained model. Thus, the relationship found in the constrained model can be potentially considered a biotic species interaction, as a non-random co-occurrence pattern exists even after accounting for host and external environmental factors (Aivelo and Norberg, 2018). In contrast, additional relationships identified in the unconstrained models are only present without controlling for host and external environmental factors. Thus, these relationships appear to be restricted to complementary or contradictory habitat requirements (host biology or the external environment) and are not strictly biotic species interactions, but rather associations shaped by their environment (Aivelo and Norberg, 2018).

It is also important to consider that flea prevalence was poorly explained by the constrained model, with fixed effects explaining less than half of the variation in flea prevalence. Furthermore, model performance was higher for the constrained model compared to the unconstrained model for all ectoparasite species except for the AUC value for flea prevalence. This suggests that the host biology and external environmental variables examined in this study are potentially poor predictors of flea prevalence. Individual ID number also explained the largest proportion of variation in flea prevalence, suggesting that there is a large degree of either unexplained variation or individual host differences in flea prevalence that remain unexplored.

It is possible that relationships identified in the unconstrained model may also be species interactions but cannot be fully discerned from the fixed effects included here (i.e. host traits, population abundance and date). For instance, flea infestation patterns can vary with external habitat components (Krasnov et al., 1998). While we did investigate variation in ectoparasite prevalence with habitat type, date and year, which may be linked to habitat components and seasonal and annual climatic factors, we did not explicitly investigate habitat components such as soil and vegetation characteristics in this study (Krasnov et al., 1998). However, habitat type and date explained very little variation in flea prevalence, suggesting missing measures of habitat components and climate may not be of concern. Given the large proportion of variation explained by individual ID number, flea prevalence may also be explained by host traits not considered in this study. For example, flea prevalence is known to vary with host foraging behaviour, range size, body size and condition (Krasnov, 2008; Krasnov et al., 2004; Perez-Orella and Schulte-Hostedde, 2005; Krasnov and Matthee, 2010). While we did examine sex and body mass, which may be indicative of range size, body size and condition (Bowers and Smith, 1979; Schulte-Hostedde et al., 2005), we did not explicitly assess the relationship between body size or condition with flea prevalence. Consequently, further investigation of factors influencing flea prevalence may provide more insight into relationships of the flea with other ectoparasite species.

Mite prevalence was largely explained by deer mouse population abundance; however, this factor was not of shared importance for flea or botfly prevalence. Generalist parasites with indirect life cycles, like the mite species in our study, may experience a stronger effect of host population-level traits compared to specialist parasites (Shatrov and Kudryashova, 2006; Lindenfors et al., 2007). Prevalence of parasites that do not share a close relationship with their hosts often varies mainly with species-level host traits, such as population abundance. Therefore, host population abundance may play a larger role in co-occurrence patterns between generalist parasite species. However, since the majority of investigated species in this study are specialists, we did not find that host abundance was a factor of shared importance between parasite species in this community.

### 4.2 Ectoparasite species interactions

The mite and botfly species had a significant and negative species interaction identified from the constrained model. This is surprising, as non-random patterns of ectoparasite co-occurrence on small mammals are frequently aggregative (Krasnov et al., 2010, Nava and Lareschi, 2014, Colombo et al., 2015, Colombo et al., 2015, but see Hoffmann et al., 2016). Antagonistic interactions tend to be due to some form of interspecific competition, leading to exclusion of one species by another (Mouillot et al., 2005). For instance, differences in body size between parasite species, such as the larger size of the botfy compared to the mite, can give one species a competitive advantage over another (Lutermann et al., 2015).

Negative interactions can be influenced by host resources, location (where parasites occur on a host), and immunology (Pedersen and Fenton, 2007; Lutermann et al., 2015). For instance, both the mite and botfly feed on host tissue and tissue fluid (Slansky, 2007; Foley et al., 2013). Parasites, compared to free-living organisms, may have very limited resource availability, restricted to size and age of their host (Lagrue and Poulin, 2008). Thus, niche overlap, such as parasite species sharing the same food resource, can lead to competition for access to host resources (Mouillot et al., 2005). In contrast, the mite species is mainly located on hosts’ ear pinnae, and occasionally on the mammae and genitalia (Ott-Conn et al., 2015), while the botfly predominantly appears in the inguinal region beneath the skin (Cogley, 1991). Therefore, a segregative pattern between the mite and botfly could be related to resource competition, but is unlikely to be influenced by competition for infestation location.

Another explanation for this antagonistic relationship between the mite and botfly is that parasites may interact via a host's immune system. For example, a low-density parasite species such as the botfly, where only a single warble was observed per deer mouse host (unpublished data), could experience excessive damage due to co-infection of a high-density species, such as the mite, where clusters of mites on hosts were often observed (unpublished data). High-density parasite species may generate a severe, non-specific immune response that has a greater effect on low-density species (Ulrich and Schmid-Hempel, 2012). In parasite networks, bottom-up (host resources) and top-down (host immune system) effects are difficult to distinguish as they share the same source (host individual) (Pedersen and Fenton, 2007). Consequently, a negative interaction between the mite and botfly may be a result of resource competition, immune system modulation, or a combination of both mechanisms.

### 4.3 Ectoparasite species associations

A negative association between the flea and mite was observed in the unconstrained model, likely due to contrasting habitat requirements (host biology or the external environment) rather than a direct species interaction (Aivelo and Norberg, 2018). This is further supported by results from experimental removal of the flea, which had no effect on mite prevalence. If the relationship between the flea and mite was a species interaction rather than an association due to habitat requirements, then we would expect a change in mite prevalence following the removal of the flea.

From variance partitioning and generalized linear mixed-effect models, it is possible that host age and sex play a role in an antagonistic association between the flea and mite. While the flea primarily infested juveniles and adults, the mite was less likely to infest adults. Furthermore, flea prevalence was highest on male deer mice, while mite prevalence was highest on females. Thus, this differentiation in host infestation patterns of each ectoparasite species may be due to intrinsic factors linked to host susceptibility rather than extrinsic factors tied to host exposure (Cattadori et al., 2006). Divergent susceptibilities of host individuals to one ectoparasite species or another may explain a negative pattern between the flea and mite in the unconstrained models*.* However, it is important to note that the fixed effects for flea prevalence explained less than half of the variation compared to random effects, suggesting that we may not have measured predictors that were of larger importance to this relationship.

Another component to consider is that the negative association between the flea and mite may also be attributed to differences in life-history traits (Hoffmann et al., 2016). While the mite is only parasitic in the larval stage and spends the rest of the life cycle off-host in the soil, the flea life cycle is more closely linked to the host (Krasnov et al., 2006b; Shatrov and Kudryashova, 2006). Specifically, fleas alternate between time spent on the host and in the nesting material of the host. Therefore, mite recruitment is likely to be dependent on external environmental conditions, while flea recruitment is likely to be linked to host biology traits (Linardi and Krasnov, 2013). This is reflected in the variance partitioning results, where host biology factors were fixed effects of greater importance for the flea, while deer mouse population abundance (which may influence encounter rates, Krasnov and Matthee, 2010) was of highest importance for the mite. Therefore, removal of fleas may reduce competition for mites. While we did not find a significant effect of flea removal, the treatment was only effective for three weeks and some host individuals had become re-infested with fleas by their next capture. Consequently, it is possible that this effect would only be clear after a longer time period where questing mite larvae can come into contact with an available host (Hoffmann et al., 2016). Therefore, the negative association between the flea and mite may be related to host traits, such as host and sex, or to parasite life-history traits, such as recruitment rates.

There was also a positive association between the flea and botfly that was observed in the unconstrained model. From variance partitioning and generalized linear mixed-effect models, host age and reproductive status were suggested to potentially influence the relationship between the flea and botfly. There was not a common pattern between these two species for infestation of different host age classes but host reproductive status may play a role in this positive relationship. Both the flea and botfly prevalence were higher on reproductively inactive hosts than reproductively active hosts. However, it is important to note that higher parasite prevalence on reproductively inactive hosts is usually due to the energetic costs that parasites can have on hosts, such as delaying development or reducing breeding production (Telfer et al., 2005; Allen and Little, 2011), rather than a greater susceptibility of reproductively inactive individuals. Thus, while a positive association between the flea and botfly is likely due to habitat requirements (host biology or external environment), it is difficult to attribute specific explanations given the factors investigated. Furthermore, we may have also missed measured predictors that were of larger importance to this relationship given the low amount of variation in flea prevalence explained by the fixed effects. The relationship may then be related to factors that were not examined in this study, such as external habitat components like soil and vegetation characteristics (Krasnov et al., 1998; Bowman, 2000), but more importantly host foraging behaviour (Slansky and Kenyon, 2002; Krasnov, 2008), and range size (Gummer et al., 1997; Krasnov et al., 2004); components known to influence both flea and botfly infestation patterns.

## 5 Conclusion

We identified a negative species association that is likely a biotic species interaction between the mite and botfly. We also identified species associations of the flea with the mite and botfly that might be due to habitat requirements, such as host characteristics, or due to parasite life-history traits. However, these results are surprising, as we expected to find mainly positive species interactions, which are much more common for ectoparasite assemblages on small mammals (Krasnov et al., 2010, Nava and Lareschi, 2014, Colombo et al., 2015, Colombo et al., 2015, but see Hoffmann et al., 2016). Antagonistic relationships can have important implications for disease management programs, as targeted reduction of one ectoparasite species could affect prevalence of other (Telfer et al., 2010). Furthermore, artificial infestation of a less harmful parasite species could be used to prevent future infestation of a more harmful species without turning to costly medication (Lello et al., 2004). Therefore, the predictive models in this study may provide insight into complex dynamics between ectoparasite species that can have ramifications for disease and parasite management. Future research should focus on experimental manipulation of the mite or botfly on deer mice to provide further insights into this ectoparasite community and whether the observed negative relationship is a true species interaction.

## Author agreement

All authors have reviewed the manuscript and approve of its submission. The manuscript is not under consideration elsewhere.

## Declaration of competing interest

The authors declare that they have no known competing financial interests or personal relationships that could have appeared to influence the work reported in this paper.
