# Supplementary material for: Parasite species co-occurrence patterns on Peromyscus: Joint species distribution modelling
Source: Int J Parasitol Parasites Wildl. 2020 Jun 14;12:199–206. doi: 10.1016/j.ijppaw.2020.04.011 (PMC7327296; doi:10.1016/j.ijppaw.2020.04.011)
Supplement: Multimedia component 1 [file mmc1.docx]

**Supplementary Material**

Figure A1. HMSC-based effect estimates of host and external environment covariates as predictors of ectoparasite species prevalence with strong statistical support (posterior probability at least 95%). Positive and negative responses are shown by red and blue entries, respectively, based on the posterior mean. DM, deer mice; RBV, southern red-backed vole; WJM, woodland jumping mouse; PA, population abundance. Population abundance of small mammal species measured as captures per 100 trap nights.

Table A1. Model performance presented as Tjur’s R^2^ and area under the receiver operating characteristic (AUC) for joint species distribution models of deer mice (n = 229 individuals).

| Model | Parasite | AUC | Tjur R^2^ |
| --- | --- | --- | --- |
| Constrained | Flea  Mite  Botfly | 0.5989  0.8159  0.8327 | 0.0222  0.2468  0.0608 |
| Unconstrained | Flea  Mite  Botfly | 0.6146  0.6885  0.6842 | 0.0154  0.0546  0.0071 |
